# Factors affecting dry eye syndrome and quality of life among college students in Korea: a cross-sectional study

**DOI:** 10.7586/jkbns.24.036

**Published:** 2025-02-17

**Authors:** Mi-Kyoung Cho, Seonhwa Yoon, Yoojin Cho, Younhye Jun, Jiseon Choi, Minyoung Ryu, Myeong Jin Kim, Ga Hyun Sung

**Affiliations:** Department of Nursing Science, Chungbuk National University, Cheongju, Korea

**Keywords:** Students, Quality of life, Dry eye syndromes, Cross-sectional studies

## Abstract

**Purpose:**

This study aimed to identify the factors affecting dry eye syndrome and quality of life (QoL) among college students.

**Methods:**

This cross-sectional study administered a descriptive survey to 184 college students across Korea. Data collection was conducted from August 27 to 28, 2024, using an online survey platform (Google Forms). The self-reported survey comprised the Health-Related Quality of Life Instrument with 8 Items scale, the Dry Eye Questionnaire 5, and the Ocular Surface Disease Index. Data analysis was performed using SPSS version 27.0.

**Results:**

The participants had a mean age of 21.37 (standard deviation 1.96) years. Regression analysis identified sleep satisfaction, vision-related functions, and dry eye symptoms as statistically significant predictors of QoL, accounting for 18.4% of the variance (F = 14.71, *p* < .001). Dry eye symptoms were significantly influenced by the use of artificial tears, vision-related functions, and environmental factors, which accounted for 41.3% of the variance (F = 43.93, *p* < .001). Additionally, significant determinants of ocular surface disease included the use of vision correction tools, the presence of ophthalmic diseases, sleep satisfaction, and dry eye symptoms. These factors explained 45.7% of the variance, confirming the model's robustness (F = 31.84, *p* < .001).

**Conclusion:**

It is necessary to identify strategies to alleviate dry eye syndrome in college students and develop comprehensive prevention and management programs to improve their QoL.

## INTRODUCTION

### 1. Background

Dry eye syndrome (DES) is a multifactorial condition resulting from damage to the ocular surface in the exposed eyelid crevices, often due to tear deficiency or excessive tear film evaporation [1]. Factors influencing DES include reduced tear production associated with aging, sex, and hormonal changes, autoimmune disorders, thyroid issues, medications like antihypertensive drugs, and environmental factors related to work and living conditions. Additionally, the modern lifestyle, particularly increased digital device use, has become a significant risk factor for DES.

The coronavirus disease 2019 pandemic, which began in late 2019, has led to a notable decrease in outdoor activities and an increase in indoor activities [2], causing a surge in the use of video viewing platforms such as YouTube, Netflix, Wave, and Tving [3]. A survey of individuals in their twenties revealed that 66.2% used video-viewing services daily or 5-6 days a week, with an average viewing duration of 57 minutes per session, highlighting both the frequency and length of use [4].

In recent years, the blend of online and offline learning has become popular, moving away from traditional instructor-centered lectures. With this shift, note-taking has transitioned from paper and pencil to digital devices like laptops, tablets, and smartphones, affecting the methods used in today's college classrooms [5]. Tablets and smartphones are now the most commonly used devices among college students, comprising 86% of their device use [6], and this increased exposure to digital media may heighten the risk of ocular strain and DES [7]. Normally, an eye blinks 10 to 16 times per minute, but it drops to five times per minute when using digital devices [8]. Viewing text on a small screen, such as a smartphone, significantly decreases blink frequency, which is crucial for maintaining normal tear production [9]. Moreover, with 75.5% of college students using both eyeglasses and contact lenses, the related exports and imports have been increasing annually [10]. Since humans receive over 90% of information visually, research has shown a significant correlation between vision and vision-related quality of life (QoL) [11]. However, studies on DES and QoL among college students are scarce.

Individuals with DES are more likely to experience ocular symptoms, predominantly tear film abnormalities, which cause discomfort and irritation. Kim et al. [1] found statistically significant associations between DES and symptoms such as eye fatigue, dryness, and a foreign body sensation in the eye. Patients reported that the most troubling symptoms involved glare and blurred vision, significantly impairing their daily activities. Additionally, severe DES symptoms correlated with lower scores on health-related QoL assessments. However, Kim and Lee [12] contend that despite negative effects such as dry eyes and reduced sleep quality resulting from extensive use of video viewing services, these activities ultimately enhance QoL. They suggest that the satisfaction derived from leisure activities like video viewing can significantly influence individual well-being [12]. It is crucial, therefore, to examine the characteristics of video viewing among college students and their correlation with DES and QoL. It is important to note that while previous epidemiological studies have shown an increase in DES prevalence among middle-aged and older adults, there has been a notable rise in cases among younger people, particularly in recent years. Yet, existing research predominantly focuses on older age groups [13]. There is a pressing need to investigate the prevalence and impact of DES among college students.

QoL is a multifaceted concept encompassing a range of domains, including physical, emotional, environmental, and objective aspects, which collectively influence an individual's overall life satisfaction [14]. In Korea, studies have identified self-esteem and self-resilience as key determinants of life satisfaction and QoL among college students [15]. Moreover, research has explored other factors such as sex, school satisfaction, academic ability, family life stress, interpersonal relationships, life stress, and depression [16]. A multidisciplinary approach that considers the subject's unique characteristics is essential to fully understand this concept. College students often experience poor sleep quality and irregular sleep-wake patterns [17]. Although the National Sleep Foundation recommends 7 to 9 hours of sleep for adults aged 18 to 25 [18], one of the studies report that 17.4% of adults sleep less than this recommended amount [19]. These sleep patterns are associated with decreased academic performance and increased work errors among college students [20]. Some studies suggest that these patterns may also affect students' adaptation to college life [21].

While it is crucial to identify the factors influencing DES and QoL, few studies have examined DES, QoL, and influencing factors specific to college students, such as digital device usage and sleep patterns. Cho et al. [22] discovered that despite a high prevalence of dry eye-related symptoms among young adults, fewer recognize themselves as having dry eye, indicating that they may be less aware of the need for treatment. Therefore, this study focuses on college students, who are at high risk for DES but often lack health awareness, with most participants being in their early 20s. The aim is to identify the characteristics of these subjects and explore the correlations and factors influencing DES and QoL to provide opportunities for early detection and management of eye health issues, thereby enhancing QoL.

### 2. Purpose

This study aims to identify the correlation and influencing factors between DES and QoL among college students. The specific objectives are as follows. First, we aim to identify the general characteristics of college students, their eye health-related characteristics, video-viewing habits, and sleep quality-related characteristics. Second, we will assess DES and QoL among college students. Third, DES and QoL will be examined in relation to the general characteristics, eye health-related characteristics, video-viewing habits, and sleep quality-related characteristics of college students. Fourth, we will identify correlations between DES and QoL in college students. Fifth, we aim to pinpoint the factors influencing both DES and QoL.

## METHODS

### 1. Study Design

This study is a cross-sectional descriptive survey aimed at determining the impact of general, eye health-related, video viewing, and sleep quality-related characteristics of college students on DES and QoL.

### 2. Participants

The study population included students aged ≥ 20 enrolled in four-year colleges and universities nationwide, selected through non-probability sampling. Students on leave were excluded due to their different lifestyles and expected lower participation rates. The sample size was calculated to include 166 subjects, based on 14 predictor variables entering the model, with a significance level of .05, a power of .90, and an effect size of .15 [23] in linear multiple regression. Data were collected from 185 subjects to allow for a 10% dropout rate. Before survey administration, the study's objectives and the data collection and analysis methods were thoroughly explained. The survey was conducted with the participants’ voluntary consent. Ultimately, 184 responses were included in the final analysis, with one excluded due to unreliable answers, such as insincere responses, misspellings, and non-responses.

### 3. Instruments

#### 1) Characteristics of the participants

First, general characteristics of college students, eye health-related characteristics, video-viewing characteristics, and sleep quality-related characteristics were identified. General characteristics included age, sex, and school year; eye health-related characteristics included vision correction devices, vision correction surgery, eye disease, and use of artificial tears; video-viewing-related characteristics included video-viewing tools, video-viewing time, video-viewing frequency, and time spent on electronic devices; and sleep quality-related characteristics included sleep duration and sleep satisfaction. Age, time spent watching videos, number of videos watched, time spent on electronics, and time spent sleeping were analyzed based on the average of the subjects' answers. Sleep satisfaction was analyzed using a 5-point Likert scale ranging from 1 for “very dissatisfied” to 5 for “very satisfied,” and the data were then categorized as representing average-or-above or below-average levels of sleep satisfaction.

#### 2) Health-Related Quality of Life Instrument with 8 Items (HINT-8)

The HINT-8 was developed by Jo [24] using qualitative and quantitative research methods to measure health-related QoL in Korea. It includes eight domains: stair climbing, pain, vitality, work, depression, memory, sleep, and happiness. Each domain is assessed on a 4-point Likert scale with responses ranging from "not at all" to "always." The index calculation formula from the HINT-8 valuation study was applied, with values ranging from 0.13 to 1. A score closer to 1 indicates better health, while a lower score indicates poorer health. The reliability, Cronbach’s ɑ of the original scale [24] was .82.

#### 3) Dry Eye Questionnaire 5 (DEQ-5)

The DEQ-5 is a condensed version of the original DEQ, comprising five items [25]. It specifically pertains to symptoms of dry eye syndrome, including eye discomfort, dryness, and excessive tearing. Each symptom's frequency is measured on a 5-point scale from "never" to "constantly," and intensity on a 6-point scale, also from "never" to "constantly." The total score is calculated by summing the frequency and intensity of symptoms, with scores ranging from 0 to 22. A score between 0 and 5 indicates normal conditions, 6 to 11 suggests DES, and 12 to 22 indicates Sjogren's syndrome [26]. A higher score reflects a more severe level of DES. The reliability, Cronbach’s alpha, of the original scale [25] was .82.

#### 4) Ocular Surface Disease Index (OSDI)

The OSDI was developed by Schiffman et al. [27] to measure and score symptoms of discomfort in the ocular surface within the past week. It includes 12 items: five related to vision function, four to ocular symptoms, and three to environmental triggers. Symptoms severity is scored on a 5-point Likert scale ranging from 0 ("never") to 4 ("always"). For items related to symptoms and environmental factors, an "N/A" option is available if not applicable. The OSDI score is calculated by multiplying the total score of the items answered by 25, then dividing by the number of items answered. The OSDI scores range from 0 to 100, where 0 to 12 is classified as normal, 13 to 22 as mild dry eye, 23 to 32 as moderate dry eye, and 33 to 100 as severe dry eye. The reliability, Cronbach’s alpha, was .92 in the original study [27] and .85 in this study.

### 4. Data collection

Data were collected through an online survey from August 27 to 28, 2024. The survey, which included 30 items, was administered via Google Forms and posted on everytime (https://everytime.kr/), a community application for college students. A Google Forms add-on, "form limiter," was used to automatically close the survey once the participant cap was reached. Before data collection, the investigator explained the study's purpose, the participants' right to withdraw, the protection of personal information, the survey’s length, and data anonymization. Participants were required to understand the study’s objectives and the method of participation, and to voluntarily give their consent before completing the survey. The average time to complete the survey was approximately 5.5 ± 0.5 minutes.

### 5. Data analysis

The data collected in this study were statistically analyzed using SPSS Statistics 27.0 (IBM Corp., Armonk, NY, USA). Statistical significance (*p*-value) was set at .05, and the reliability of the DES and QoL scales was assessed using Cronbach's alpha. The specific methods of analysis were as follows. Subject characteristics, dry eye-related characteristics, and QoL-related characteristics of college students were described using means, standard deviations, and ranges. The relationship between DES and QoL in relation to subject characteristics was explored. This analysis included conducting an independent t-test and one-way analysis of variance after verifying normality with the Shapiro-Wilk test. Post hoc comparisons were performed using Scheffe's test. The relationship between DES and QoL was further analyzed using the Pearson correlation coefficient. The effects on DES and QoL were examined using multiple linear regression. Statistical inference was conducted at a significance level of *p* <.05.

### 6. Ethical considerations

This study was conducted after receiving approval from the Chungbuk National University IRB Committee (CBNU-2024-A-0044). It included information on the study’s background, purpose, procedures, anonymizing collected data, and using data solely for research purposes. It stated the participants' rights to refuse participation or withdraw at any time without penalty. Participants had to consent via an online consent form before the survey. The investigator's contact information (telephone number and email address), was provided on the start page of the survey. Additionally, responses from those who declined to participate were excluded from the final analysis. Upon completion, the first 184 participants were awarded an online gift certificate for a café. It was explicitly stated that phone numbers collected for sending the coupons would only be used for that purpose and would be discarded immediately after use.

## RESULTS

### 1. Characteristics of the participants

Regarding the participants' general characteristics, the average age was 21.37 (standard deviation = 1.96) years, with female comprising 135 (73.4%) of the sample. Regarding grade, 62 (33.7%) were third-year students. For vision correction tool, 82 (44.6%) used glasses, and 30 (16.3%) used contact lenses. Regarding vision correction surgery, 16 (8.7%) underwent Smilelasik, 13 (7.1%) had lasek, and three (1.6%) had lasik. Eighteen participants (9.8%) reported having an eye disease. Smartphones were the most commonly used device for watching videos, utilized by 117 (63.6%) participants. Additionally, 100 (54.3%) used artificial tears. The average video watching time was 3.58 ± 1.96 hours, and the average video watching frequency was 5.95 ± 4.27 times. The average electric device usage time was 7.08 ± 2.47 hours, and the average sleep time was 7.27 ± 1.36 hours. In terms of sleep satisfaction, 144 participants (78.3%) rated their sleep as average or above, while 40 (21.7%) rated it below average (Table 1).

### 2. QoL and DES

The QoL scores closer to 1 indicate better health status. In this study, the score was 0.83 ± 0.07, reflecting good health [24]. For the dry eye symptoms, a total score of less than 6 indicates normal eye conditions, 6 to 11 suggests DES, and 12 or more points to Sjogren's syndrome [28]. In this study, the average score was 7.16 ± 4.51, categorizing the condition as DES. For the ocular surface diseases, a score range of 0 to 12 is considered normal, 13 to 22 as mild dry eye, 23 to 32 as moderate DES, and 33 to 100 as severe DES [29]. The average score in this study was 17.79 ± 14.64, placing it within the mild DES category (Table 2).

### 3. Differences between QoL and DES according to participants’ characteristics

The QoL scores were significantly influenced by participant characteristics such as sex (t = 2.24, *p* = .027), type of vision correction tool (F = 5.26, *p* = .006), use of artificial tears (t = 2.19, *p* = .030), sleep time (t = −2.54, *p* = .012), and sleep satisfaction (F = −4.12, *p* < .001). Specifically, scores were higher among men compared to women, and higher among those using glasses or no vision correction than those using lenses. Higher QoL scores were also observed in participants who did not use artificial tears, had average or above sleep duration, and had average or above sleep satisfaction. Other characteristics did not show significant differences.

The dry eye symptoms showed significant differences for sex (t = −2.16, *p* = .032), type of vision correction tool (F = 4.25, *p* = .016), presence of eye disease (t = −2.28, *p* = .024), use of artificial tears (t = −6.57, *p* < .001), type of video equipment (F = 3.23, *p* = .024), and sleep satisfaction (F = 3.79, *p* < .001). The dry eye symptoms were higher among women than men, higher among those with lenses than those with glasses or without vision correction, higher among those with an eye condition and those who used artificial tears compared to those who did not, and higher among those who watched videos on televisions compared to those using smartphones, tablets, and computers. Scores were also higher when sleep satisfaction was less than moderate. Other characteristics were not significant.

The ocular surface diseases revealed significant differences for sex (t = −2.92, *p* = .004), type of vision correction device (F = 8.70, *p* < .001), presence of eye disease (t = −5.01, *p* < .001), use of artificial tears (t = −3.63, *p* < .001), and sleep satisfaction (F = 4.65, *p* < .001). The ocular surface diseases scores were higher among women than men, higher among those with lenses compared to those with glasses or without vision correction, and higher among those with an eye condition and those using artificial tears than those who did not. The ocular surface diseases scores were also higher for participants watching videos on television and when sleep satisfaction was less than moderate. Other characteristics did not show significant differences (Table 3).

### 4. Correlations between variables

The QoL had a significant weak negative correlation with the dry eye symptoms (r = −.34, *p* <.001) and the ocular surface diseases (r = −.34, *p* <.001). This indicated that DES accounted for 11.6% of the variance in QoL. The effect size calculated from this study's correlation coefficient between DES and QoL was 0.3, a small effect size (Table 4).

### 5. Factors affecting the QoL and DES

A stepwise multiple regression analysis was used and variables were entered into the model when the significance level was ≤ .050 and removed when it was ≥ .100. Independent variables were set based on the characteristics and variables of the constructed model, categorical variables were dummied, and continuous variables were entered as they were. Results showed that the variance inflation factor values ranged from 1.06 to 1.37 (Ref. < 10), and the tolerance values ranged from 0.73 to 0.94 (Ref. > 0.1), indicating that the independent variables are independent and without multicollinearity problems. Furthermore, the Durbin-Watson values ranged from 1.83 to 2.35, all close to 2 and not close to 0 or 4, indicative of the independence of the residuals (Table 5).

Factors impacting QoL included sleep satisfaction, vision-related function on the ocular surface diseases, and the dry eye symptoms. Higher sleep satisfaction was associated with improved QoL among participants. Conversely, higher the ocular surface diseases scores for vision-related function and higher the dry eye symptoms scores for DES were linked to a lower QoL. The explanatory power of this model was 18.4%, with the regression model proving statistically significant (F = 14.71, *p* < .001).

Three factors influenced the dry eye symptoms: the use of artificial tears, vision-related function, and environmental factors. Usage of artificial tears, alongside higher scores on the two mentioned OSDI items, corresponded with increased the dry eye symptoms scores. These three factors explained 41.3% of the variance in the dry eye symptoms score, and the regression model was found to be statistically significant (F = 43.93, *p* < .001).

The factors affecting the ocular surface diseases score included types of vision correction devices, presence of eye disease, sleep satisfaction, and the dry eye symptoms score. The ocular surface diseases scores were higher for participants using glasses or lenses compared to those without any vision correction devices, and for those with an eye condition compared to those without. Lower sleep satisfaction and higher the dry eye symptoms scores were associated with higher the ocular surface diseases scores. The explanatory power for this model was 45.7%, and the regression model was statistically significant (F = 31.84, *p* <.001).

## DISCUSSION

This study explores the relationship between QoL and DES in college students. The propensity for DES in women, previously reported in several studies [30,31], was also observed here, with female participants displaying higher the dry eye symptoms and ocular surface diseases scores compared to male participants. Additionally, participants who viewed videos on televisions reported higher dry eye symptoms and ocular surface diseases scores than those using smartphones, tablets, or laptops. This aligns with findings from previous research, which indicated that tear breakup time decreased after television viewing, suggesting a link to DES [32]. However, as only 2.2% of the students in this study watched TV, a controlled follow-up study examining the usage of computers, smartphones, and televisions is warranted.

From a pathophysiological perspective, hypoxia during lens wear leads to a shift from aerobic to anaerobic metabolism in the cornea, resulting in the accumulation of carbon dioxide and lactic acid. This process causes the cornea to become acidic and edematous [33]. Consistent with previous findings that contact lens wearers under the age of 50 are 2.39 times more likely to develop DES compared to non-lens wearers [34], this study found that participants with contact lenses had elevated dry eye symptoms and ocular surface diseases scores relative to those without vision correction and those with glasses. However, glasses were also identified as a factor influencing DES. Previous research indicated high concurrent use of glasses and contact lenses, with 75.5% of individuals using both [10]. This study did not investigate the combined use of glasses and contact lenses, highlighting the need for further research.

In this study, the history of corrective eye surgery was not significantly associated with dry eye, aligning with previous findings [29] that reported no difference in the prevalence of dry eye between individuals who had undergone corrective eye surgery and those who had not. The presence of eye conditions, such as vision impairment and keratitis, was significantly associated with dry eye, with affected individuals showing higher dry eye symptoms and ocular surface diseases scores. This is consistent with research suggesting that conditions like blepharitis could lead to lacrimal gland dysfunction [35]. Additionally, a positive correlation between the use of artificial tears and DES was observed, which echoes findings from another study [29] that noted a higher prevalence of DES among users of artificial tears.

In this study, less than moderate sleep satisfaction was associated with higher dry eye symptoms and ocular surface diseases scores, confirming sleep satisfaction as a significant predictor of the ocular surface diseases. This finding is consistent with previous studies indicating that reduced sleep increases stress hormones such as cortisol, epinephrine, and norepinephrine, and a decrease in parasympathetic nerves [36], which in turn decreases tear production, contributing to the vicious cycle of DES [30]. Excessive use of digital devices, through blue light exposure, inhibits the secretion of melatonin and leads to incomplete blinking and uneven distribution of tears, which triggers dry eye symptoms. Dry eye causes ocular discomfort and may lead to discomfort during sleep, while simultaneously disrupting the autonomic nervous system, thereby worsening sleep quality [37]. As previously noted, the deterioration in sleep quality exacerbates dry eye symptoms, creating a vicious cycle.

Prior research has shown that in environments with low relative humidity, such as air-conditioned rooms or windy outdoor settings, the water vapor pressure gradient between the eye surface and the surrounding environment increases, leading to greater tear evaporation [38]. This study found that environmental factors also significantly affected the dry eye symptoms score, aligning with these findings. Previous studies in university settings have reported the dry eye symptoms and ocular surface diseases scores of 7.5 ± 4.8 and 20.3 ± 17.4, respectively, classifying both within the DES category [28,29]. According to statistics from the Health Insurance Review & Assessment Service's big data opening system, Korea's number of patients with DES reached 2.45 million as of 2020, steadily increasing yearly. Among them, those in their 60s accounted for the highest percentage, 19.4% of the population, their 50s accounted for 19.1%, and college students, predominantly in their early 20s, accounted for 11.6% of the cases [39]. Another study from Korea found a DES prevalence of about 12.8% (62 out of 486 patients) [7]. Comparatively, this study recorded a DES rate of 40% (73 out of 184 participants), which is significantly higher than previous findings. Conducted in August during the summer months when air conditioners were frequently used, it is possible that environmental factors related to air conditioning influenced the high DES rates observed. College students mainly study indoors with air conditioners and heaters, and the frequency of exposure to digital screens through the use of digital devices during class hours is increasing due to the social system where there are almost no restrictions on Internet access [26]. If further research is conducted on the degree of dry eye according to learning media and the degree of dry eye according to learning environment, it will help establish measures to prevent dry eye in college students. In this study, the vision-related function items of the ocular surface diseases were found to be significantly and positively correlated with the dry eye symptoms. This correlation is supported by recent findings [40] indicating that many individuals with low vision experience DES as a comorbidity. Moreover, the vision-related function items of the ocular surface diseases have been identified as significant predictors of QoL, with research showing that decreased visual acuity is associated with reduced QoL in areas such as motor skills, activities of daily living, and pain/discomfort, primarily due to activity limitations caused by low vision [41]. Consistent with prior research, this study found that poor and blurry vision and sore or stinging eyes were linked to a lower QoL.

Additionally, the dry eye symptoms showed a significant negative correlation with QoL. One study reported that DES-related eye symptoms were associated with lower scores on health-related QoL measures [1]. An analysis of QoL differences by sex revealed that, as found in previous research [42] and corroborated by this study, women generally reported lower QoL than men. This aligns with the observation in this study that more severe DES symptoms, indicated by higher the ocular surface diseases and dry eye symptoms scores, are associated with lower QoL. Regarding vision correction devices, teenage contact lens wearers reported positive impacts on appearance, satisfaction, activities, and peer perceptions compared to glasses wearers [43]. Conversely, a study of individuals in their 20s and 30s (69.56%) found that symptoms such as dryness, foreign body sensation, blurred vision, and redness were commonly reported discomforts associated with wearing contact lenses [44]. Furthermore, a study focusing on individuals in their 20s observed that those who wore glasses reported a higher QoL than those wearing contact lenses [13]. Similarly, this study confirmed that those who wore glasses had a higher QoL than contact lens wearers. The higher prevalence of DES among lens wearers noted in this study may also be relevant. The use of artificial tears was significantly positively correlated with both dry eye symptoms and ocular surface diseases scores and was associated with lower QoL. This likely reflects that the use of artificial tears is correlated with DES, which in turn is related to QoL. However, using artificial tears could not be interpreted as a direct predictor of improved QoL, contrasting with a previous study [45] that demonstrated how artificial tears increased tear film stability and reduced perceived discomfort in both normal and DES-afflicted eyes.

The study identified sleep satisfaction, vision-related function on the ocular surface diseases, and the dry eye symptoms score as significant factors affecting QoL. Firstly, sleep satisfaction was found to have a positive correlation with QoL, echoing research that indicates adequate sleep improves mood, boosts immune function, aids weight management, enhances mental health, and reduces the risk of many chronic diseases [46]. Consistent with studies showing that sleep deprivation adversely affects enjoyment of life and daily functioning [47], this study also revealed that longer sleep duration correlated with higher life satisfaction. However, the direct interpretation of sleep duration as a QoL factor remained inconclusive. A study involving medical students found a significant relationship between QoL and sleep duration [48]. Although the average weekday sleep duration for participants in this study was 6 hours and 18 minutes, those with longer sleep durations reported better QoL. Nevertheless, considering the World Sleep Society's recommendation of 7 to 9 hours of sleep, the average sleep duration in this study was 7 hours and 27 minutes, which is higher than in previous studies, indicating a possible weaker association with QoL.

This study's QoL scored 0.83 ± 0.07, suggesting good health. A previous study in Korea measured life satisfaction among university students using three items developed by Kim et al. [49] and reported a standardized score of 0.70 ± 0.03, indicating high satisfaction. Similarly, a study in Israel measured life satisfaction with a single-scale measure and reported a standardized score of 0.75 ± 0.13, also indicating high satisfaction, which aligns with the findings of this study [50].

Furthermore, healthy eyes and clear vision are crucial for information acquisition and adapting to lifestyle changes in the modern world [1]. A previous Korean study on college students found that those with DES symptoms had lower scores in health-related QoL domains and those with more severe dry eye experienced significantly greater work productivity loss and impairment in daily activities compared to those with milder DES [7]. This aligns with the current study’s findings, confirming that DES is significantly negatively associated with QoL.

Based on these findings, the need for further research is evident. This study highlighted that sleep dissatisfaction significantly reduces QoL among college students. Consequently, future research should consider implementing sleep education programs and campaigns to enhance sleep habits, aiming to improve their QoL. Given that the subjects of this study were predominantly college students, who often use digital devices for extended periods due to academic demands, it is not surprising that 40% of participants were affected by DES, which was found to significantly negatively impact their QoL. Thus, further research into preventive and management strategies for DES is crucial to enhance QoL in this demographic. Furthermore, sleep satisfaction, DES, and vision-related issues were closely linked not only to the physical health of college students but also to their mental and social well-being. Follow-up studies should include a comprehensive health promotion program that raises overall health awareness. It would be beneficial to investigate how various health factors, such as mental health, nutrition, and physical activity, influence eye health and sleep quality. The insights gained could be used to develop a holistic health promotion strategy for college students.

This study used a self-reported survey and has the following limitations. First, self-reported surveys rely on the subjective evaluation of respondents, so there is a possibility of recall bias or social desirability bias. These biases involve the risk that respondents may exaggerate or underreport their experiences or behaviors, which may affect the accuracy and reliability of the data. Second, because of the nature of the cross-sectional design, only data collected at a specific point in time are analyzed, so changes over time or causal relationships cannot be sufficiently explained.

## CONCLUSION

This descriptive survey study assessed the impact of DES and QoL among 184 undergraduate students from four-year universities nationwide. The findings indicated that QoL was higher among men, those who wear glasses or do not use vision correction devices, those who do not use artificial tears, and those with average or better sleep duration and satisfaction. Factors associated with DES included being female, watching videos on television, wearing glasses or lenses, having an eye disease, using artificial tears, and having less than moderate sleep satisfaction. Additionally, factors affecting QoL were identified as sleep satisfaction, vision-related function on the ocular surface disease, and the dry eye symptom score, underscoring the impact of sleep satisfaction and DES on QoL. Given these findings, it is crucial to identify strategies to alleviate DES in college students and develop comprehensive prevention and management programs to enhance their QoL.

## Figures and Tables

**Table 1.** Characteristics of the Participants Related to QoL and DES (N = 184)

Characteristics	Categories	n	%	M ± SD	Min ~ Max
Age (yr)	< 21.37	104	56.5	21.37 ± 1.96	18.00 ~ 28.00
	≥ 21.37	80	43.5		
Sex	Men	49	26.6		
	Women	135	73.4		
Year in school	1^st^	50	27.2		
	2^nd^	37	20.1		
	3^rd^	62	33.7		
	≥ 4^th^	35	19.0		
Vision correction tool	Glasses	82	44.6		
	Lens	30	16.3		
	Not available	72	39.1		
Vision correction surgery	Lasik	3	1.6		
	Lasek	13	7.1		
	Smilelasik	16	8.7		
	Implantable contact lens	2	1.1		
	Not available	150	81.5		
Eye diseases	No	166	90.2		
	Yes	18	9.8		
Artificial tears	No	84	45.7		
	Yes	100	54.3		
Video equipment	Smartphone	117	63.6		
	Tablet	51	27.7		
	Desktop	12	6.5		
	Television	4	2.2		
Video watching time (hr)	< 3.58	109	59.2	3.58 ± 1.96	0.00 ~ 14.50
	≥ 3.58	75	40.8		
Video watching (frequency)	< 5.95	109	59.2	5.95 ± 4.27	0.50 ~ 29.00
	≥ 5.95	75	40.8		
Electronic device usage time (hr)	< 7.08	121	65.8	7.08 ± 2.47	2.00 ~ 16.50
	≥ 7.08	63	34.2		
Sleep time (hr)	< 7.27	85	46.2	7.27 ± 1.36	3.00 ~ 12.00
	≥ 7.27	99	53.8		
Sleep satisfaction	< 3.38	40	21.7	3.38 ± 1.01	1.00 ~ 5.00
	≥ 3.38	144	78.3		

QoL= Quality of life; DES = Dry eye syndrome; M = Mean; SD = Standard deviation; Min = Minimum; Max = Maximum; Lasik = Laser in situ keratomileusis; Lasek = Laser epithelial keratomileusis.

**Table 2.** Descriptive Statistics for QoL and DES (N = 184)

Variables	Item	M ± SD	Min ~ Max
QoL	8	0.83 ± 0.07	0.50 ~ 0.93
Climbing stairs	1	0.92 ± 0.01	0.86 ~ 0.93
Pain	1	0.90 ± 0.03	0.77 ~ 0.93
Vitality	1	0.91 ± 0.01	0.86 ~ 0.93
Working	1	0.93 ± 0.00	0.90 ~ 0.93
Depression	1	0.92 ± 0.01	0.83 ~ 0.93
Memories	1	0.92 ± 0.02	0.87 ~ 0.93
Sleep	1	0.92 ± 0.01	0.84 ~ 0.93
Happiness	1	0.92 ± 0.03	0.85 ~ 0.93
Dry eye symptoms	5	7.16 ± 4.51	0.00 ~ 20.00
Ocular surface diseases	12	17.79 ± 14.64	0.00 ~ 64.60
Vision-related function	5	21.98 ± 17.61	0.00 ~ 80.00
Ocular symptoms	4	11.28 ± 15.97	0.00 ~ 87.50
Environmental triggers	3	19.43 ± 21.24	0.00 ~ 100.00

QoL= Quality of life; DES = Dry eye syndrome; M = Mean; SD = Standard deviation; Min = Minimum; Max = Maximum. DES encompasses dry eye symptoms and ocular surface diseases.

**Table 3.** Difference in QoL and DES According to Participants’ Characteristics (N = 184)

Characteristics		QoL		Dry eye symptoms		Ocular surface diseases	
		M ± SD	F or t (*p*) (Scheffe)	M ± SD	F or t (*p*) (Scheffe)	M ± SD	F or t (*p*) (Scheffe)
Age (yr)	< 21.37	0.82 ± 0.08	−0.88 (.382)	7.30 ± 4.40	0.46 (.645)	20.50 ± 15.30	−0.60 (.553)
	≥ 21.37	0.83 ± 0.63		6.99 ± 4.68		21.89 ± 16.29	
Sex	Men	0.85 ± 0.70	2.24 (.027)	5.98 ± 4.52	−2.16 (.032)	15.60 ± 13.97	−2.92 (.004)
	Women	0.82 ± 0.07		7.59 ± 4.45		23.10 ± 15.87	
Year in school	1^st^	0.84 ± 0.07	0.71 (.548)	6.18 ± 4.16	1.53 (.208)	20.42 ± 17.10	0.93 (.430)
	2^nd ^	0.82 ± 0.09		7.32 ± 4.22		18.06 ± 13.18	
	3^rd^	0.82 ± 0.07		7.98 ± 4.43		23.37 ± 15.12	
	≥ 4^th^	0.83 ± 0.06		6.94 ± 5.27		21.31 ± 17.11	
Vision correction tool	Glasses^a^	0.84 ± 0.06	5.26 (.006)	6.38 ± 4.52	4.25 (.016)	21.58 ± 16.18	8.70 (<. 001)
	Lens^b^	0.79 ± 0.10	(a,c > b)	9.13 ± 4.22	(a,c < b)	30.34 ± 12.28	(a,c < b)
	Not available^c^	0.83 ± 0.72		7.24 ± 4.43		16.72 ± 14.82	
Vision correction surgery	Lasik	0.77 ± 0.11	0.84 (.502)	8.00 ± 7.00	0.60 (.662)	30.24 ± 28.94	0.56 (.689)
	Lasek	0.82 ± 0.08		6.85 ± 5.27		18.07 ± 12.43	
	Smilelasik	0.82 ± 0.09		8.75 ± 3.42		20.26 ± 15.31	
	Implantable contact lens	0.89 ± 0.01		6.00 ± 2.83		11.94 ± 2.75	
	Not available	0.83 ± 0.72		7.02 ± 4.54		21.40 ± 15.87	
Eye diseases	No	0.83 ± 0.07	1.27 (.207)	6.92 ± 4.42	−2.28 (.024)	19.31 ± 14.80	−5.01 (< .001)
	Yes	0.81 ± 0.09		9.44 ± 4.88		37.66 ± 14.42	
Artificial tears	No	0.84 ± 0.07	2.19 (.030)	5.01 ± 3.87	−6.57 (< .001)	16.66 ± 14.48	−3.63 (< .001)
	Yes	0.82 ± 0.08		8.97 ± 4.23		24.84 ± 15.80	
Video equipment used	Smartphone^a^	0.83 ± 0.07	1.38 (.251)	6.69 ± 4.39	3.23 (.024)	20.17 ± 15.95	2.40 (.069)
					(a,b,c < d)		
	Tablet^b^	0.82 ± 0.08		7.45 ± 4.35		21.98 ± 14.66	
	Desktop^c^	0.83 ± 0.10		8.58 ± 5.14		19.95 ± 13.73	
	Television^d^	0.77 ± 0.08		13.00 ± 4.83		40.96 ± 18.99	
Video watching time (hr)	< 3.58	0.83 ± 0.75	−0.12 (.908)	7.08 ± 4.29	−0.29 (.772)	21.02 ± 16.06	−0.87 (.931)
	≥ 3.58	0.83 ± 0.74		7.28 ± 4.85		21.23 ± 15.29	
Video watching (frequency)	< 5.95	0.83 ± 0.79	0.24 (.814)	7.49 ± 4.42	1.17 (.243)	22.10 ± 15.47	1.03 (.304)
	≥ 5.95	0.83 ± 0.68		6.69 ± 4.63		19.67 ± 16.04	
Electronic device usage time (hr)	< 7.08	0.82 ± 0.08	−1.28 (.201)	7.30 ± 4.41	0.60 (.577)	21.84 ± 15.83	0.88 (.378)
	≥ 7.08	0.84 ± 0.07		6.90 ± 4.74		19.69 ± 15.50	
Sleep time (hr)	< 7.27	0.81 ± 0.80	−2.54 (.012)	7.45 ± 4.30	0.79 (.431)	22.79 ± 15.80	1.36 (.177)
	≥ 7.27	0.84 ± 0.68		6.92 ± 4.70		19.66 ± 15.56	
Sleep satisfaction	< 3.38	0.79 ± 0.08	-4.12 (< .001)	9.48 ± 4.20	3.79 (< .001)	30.78 ± 18.69	4.65 (< .001)
	≥ 3.38	0.84 ± 0.07		6.52 ± 4.40		18.42 ± 13.67	

QoL= Quality of life; DES = Dry eye syndrome; M = Mean; SD = Standard deviation. Post hoc: Scheffe’s test; Superscripts a, b, and c are groups for Scheffe’s test. DES encompasses dry eye symptoms and ocular surface diseases.

**Table 4.** Correlations between QoL and DES (N = 184)

Variables		QoL	Dry eye symptoms	Ocular surface diseases			
				Total	Vision-related function	Ocular symptoms	Environmental triggers
r (*p*)							
QoL		1	−.34 (< .001)	−.34 (< .001)	−.34 (< .001)	−.17 (.019)	−.29 (< .001)
Dry eye symptoms		-	1	.57 (< .001)	.48 (< .001)	.39 (< .001)	.53 (< .001)
Ocular surface diseases	Total	-	-	1	.87 (< .001)	.79 (< .001)	.77 (< .001)
	Vision-related function	-	-	-	1	.53 (< .001)	.49 (< .001)
	Ocular symptoms	-	-	-	-	1	.44 (< .001)

QoL = Quality of life; DES = Dry eye syndrome. DES encompasses dry eye symptoms and ocular surface diseases.

**Table 5.** Factors Affecting QoL and DES (N = 184)

Variables	QoL			Dry eye symptoms			Ocular surface diseases		
	B (SE)	β	t (*p*)	B (SE)	β	t (*p*)	B (SE)	β	t (*p*)
(Constant)	0.81 (0.02)		36.06 (< .001)	2.95 (0.46)		6.37 (< .001)	14.66 (3.59)		4.08 (< .001)
Vision correction tool (ref. = not available)									
Glass							5.43 (1.77)	0.18	3.07 (.002)
Lens							5.73 (2.41)	0.14	2.38 (.018)
Eye diseases (ref. = no)									
Yes							10.08 (2.75)	0.21	3.66 (< .001)
Artificial tears				2.60 (0.54)	0.29	4.84 (< .001)			
Sleep satisfaction	0.02 (0.01)	0.21	3.01 (.003)				−3.61 (0.83)	−0.25	−4.36 (< .001)
Dry eye symptoms	0.00 (0.00)	−0.19	−2.46 (.015)				1.53 (0.19)	0.47	8.12 (< .001)
Ocular surface diseases									
Vision-related function	0.00 (0.00)	−0.19	−2.43 (.016)	0.07 (0.02)	0.26	3.98 (< .001)			
Environmental triggers				0.07 (0.01)	0.32	4.88 (< .001)			
F(*p*)	14.71 (< .001)			43.93 (< .001)			31.84 (< .001)		
adj. *R*^2^(%)	18.4			41.3			45.7		
Tolerance	0.74 ~ 0.89			0.73 ~ 0.91			0.80 ~ 0.94		
VIF	1.12 ~ 1.35			1.10 ~ 1.37			1.06 ~ 1.25		
Durbin-Watson	1.83			1.84			2.35		

QoL = Quality of life; DES = Dry eye syndrome; SE = Standard error; ref. = Reference; VIF = Variance inflation factor; adj *R*^2^. = Adjusted R-squared. DES encompasses dry eye symptoms and ocular surface diseases.

## Data Availability

The data that support the findings of this study are available from the corresponding author upon reasonable request.
